# Randomized control trials using a tablet formulation of hyperimmune bovine colostrum to prevent diarrhea caused by enterotoxigenic *Escherichia coli* in volunteers

**DOI:** 10.3109/00365521.2011.574726

**Published:** 2011-04-29

**Authors:** Wlodzimierz Otto, Boguslaw Najnigier, Teodor Stelmasiak, Roy M Robins-Browne

**Affiliations:** 1 Department of General and Liver Surgery, Medical University of Warsaw, Warsaw, Poland; 2 Glutagen Pty Ltd, Maribyrnong, Victoria, Australia; 3 Department of Microbiology and Immunology, The University of Melbourne, Victoria, Australia; 4 Murdoch Childrens Research Institute, Royal Children's Hospital, Parkville, Victoria, Australia

**Keywords:** Enterotoxigenic *Escherichia coli*, hyperimmune bovine colostrum, travelers' diarrhea, volunteers

## Abstract

***Objective***. Enterotoxigenic *Escherichia coli* (ETEC) is the leading cause of travelers' diarrhea. The aim of this study was to investigate the ability of a powdered extract of hyperimmune bovine colostrum to protect against diarrhea in volunteers challenged with ETEC. ***Materials and methods***. Tablets were manufactured from a colostrum extract from cattle immunized with 14 ETEC strains, including serogroup O78. Two separate randomized, double-blind, placebo-controlled trials involving 90 healthy adult volunteers were performed to investigate the ability of different tablet formulations to protect against diarrhea following an oral challenge with an O78 ETEC strain. ***Results***. The first study with 30 participants evaluated the efficacy of tablets, containing 400 mg of colostrum protein, taken thrice daily with bicarbonate buffer. This regimen conferred 90.9% protection against diarrhea in the group receiving the active preparation compared with the placebo group (*p* = 0.0005). The second study examined the efficacy of tablets containing 400 mg colostrum protein given with buffer (83.3% protection;*p* = 0.0004) or without buffer (76.7% protection;*p* =0.007), and tablets containing 200 mg colostrum protein given without buffer (58.3% protection; *p* = 0.02), compared with placebo. The difference between buffered and unbuffered treatments was not significant (*p* > 0.1). ***Conclusions***. Active tablet formulations were significantly more effective than placebo in protecting volunteers against the development of diarrhea caused by ETEC. These results suggest that administration of a tablet formulation of hyperimmune bovine colostrum containing antibodies against ETEC strains may reduce the risk of travelers' diarrhea.

## Introduction

Diarrhea is a common affliction of travelers especially, those who visit less-developed countries [1,2]. Between 20% and 70% of visitors to such countries suffer from diarrhea, which in approximately 50% of cases is caused by strains of enterotoxigenic *Escherichia coli* (ETEC) [1–4]. Currently, the management of travelers' diarrhea is based on rehydration and the relief of symptoms. Prophylactic measures are of questionable efficacy [5,6], and antimicrobial agents are not widely used because of the risk of adverse side-effects and reduced effectiveness due to the emergence of antibiotic-resistant bacteria [1]. As travelers' diarrhea is mostly seen as a nuisance, rather than life-threatening, preven-tative interventions, especially drugs, should be free from unwanted side-effects.

Our investigations of hyperimmune bovine colostrum as a prophylactic measure are based on the protection provided by colostrum and breast milk to infants of various animal species [7,8]. Previous work in this area has shown that the ingestion of antibodies derived from hyperimmune colostrum or milk can prevent the symptoms of infection with ETEC [9]. As ETEC infects the small intestine, however, the protective antibodies in colostrum must traverse the acid environment of the stomach intact and reach their site of action in adequate concentrations [10]. For this reason, the effectiveness of passive immunization with bovine antibodies requires the ingestion of large quantities of antibodies or the co-administration of buffering agents, which paradoxically may increase susceptibility to various infections by reducing the efficacy of the gastric acid barrier [10,11].

The aim of this study was to investigate the ability of a novel preparation of a powdered extract of hyper-immune bovine colostrum, with some innate acid resistance, to protect against the development of diarrhea after challenge of volunteers with ETEC.

## Materials and methods

### Preparation of hyperimmune bovine colostrum powder

The bovine colostrum powder (BCP) used for this study was made from the colostrum of dairy cows that had been immunized with antigens derived from 14 ETEC strains belonging to O-serogroups, O6, O8, O15, O20, O25, O27, O63, O78, O114, O115, O128, O148, O153 and O159 [9]. The strains used to manufacture the vaccine were selected because of their recognized role in travelers' diarrhea, and because they express common O-, H- and colonization factor antigens of human ETEC [3,9,12]. The colonization factor antigens carried by these strains collectively were CFA/I, CS1, CS2, CS3, CS4, CS5, CS6, CS7, CS12, CS14 and CS17. The vaccine was prepared in accordance with Australian Patent number 2004216920 as follows. Each ETEC strain was cultured on several CFA agar plates at 37° C overnight [13]. The bacteria were then suspended in ∼2 ml of 0.1 M phosphate buffered saline (PBS, pH 7.2) containing 0.05% sodium azide. The suspension was vortexed vigorously to disperse any clumps and then homogenized 15 times for 1-min periods, interspersed with 1 min of cooling in an ice bath. The homogenized preparation was centrifuged at 12,000 × *g* for 20 min at 4°C. The supernatant was collected and precipitated with 20% ammonium sulphate, allowed to stand for 60 min at 4°C and then re-centrifuged as above. The supernatant from this centrifugation was precipitated with 40% ammonium sulphate and re-centrifuged. The resulting pellet was suspended in 0.05% PBS and dialyzed against 250–1000 volumes of cold PBS using a 3.5-kDa molecular-weight-cut-off membrane. The protein content of the final dialysate was adjusted to 1 mg/ml and stored at −20°C in PBS containing 0.3% formalin. The vaccine preparation containing equal quantities of dialysate from each of the 14 ETEC strains was thoroughly emulsified with an equal volume of adjuvant (Montanide ISA 206; Seppic, France) and checked for sterility before use. Two milliliters of the vaccine was administered by intramuscular injection to adult pregnant dairy cows 5 times over 10 weeks. Dairy cattle from Australia were used as Australia is approved by the European Union as being free of bovine spongiform encephalopathy. The protocol for the inoculation of cows was approved by the Office of the Chief Veterinary Officer, Victoria, Australia, and conformed with the “Australian Code of Practice for the Care and Use of Animals for Scientific Purposes” published by the Australian National Health and Medical Research Council.

Colostrum from the first milking after calving was collected into refrigerated milk vats and then frozen for further processing. This included defatting, pasteurization and ultrafiltration using equipment, work practices and hygiene standards conforming to approved dairy factory codes of practice used in Australia [14]. The liquid concentrate was freeze-dried to form the powder, referred to as BCP. Tablets were made from BCP in accordance with Good Manufacturing Practice and safety standards stipulated by the Therapeutic Goods Administration of Australia. Each tablet contained 200 mg of protein of BCP. Placebo tablets were manufactured to the same standards, but contained lactose instead of BCP.

### Assay for antibodies

Tablets containing BCP were assayed by solid phase enzyme immunoassay (EIA) for antibodies to *E. coli* serogroup O78 as follows: 5 × 10^7^ heat-killed *E. coli* O78 cells in 100 µl carbonate–bicarbonate coating buffer were dispensed into the wells of a 96-well Maxisorp Immuno-plate (Nunc, Roskilde, Denmark) and left at 4°C overnight. Plates were washed 6 times in PBS-0.05% Tween buffer, comprising 137 mM NaCl, 8 mM KCl, 1.5 mM KH_2_PO4, and 8 mM Na_2_HPO_4_ at pH 7.4. One hundred microlitres of each test sample, diluted in PBS-Tween containing 12 mg/ml casein, was added to each well and incubated at 37°C for 2 h. Plates were washed 6 times in PBS-Tween buffer, after which 100 µl of goat anti-bovine IgG-peroxidase conjugate (Southern Biotechnology Associates, Birmingham, AL, USA), diluted 1:4000 in PBS-Tween-casein, was added to each well. Plates were incubated for 1 h at 37°C and washed 6 times. One hundred microlitres of peroxidase sub-strate (Kirkegaard and Perry Lab. Inc., Gaithersburg, MD, USA) was then added to each well and left at room temperature until color developed. The reaction was stopped by the addition of 2 M sulphuric acid, and the plates were read in a Diagnostics Pasteur LP400 plate reader (Sanofi, Marnes-la-Coquette, France) at 450 nm. Results were determined as the mean net OD (after subtraction of the blank reaction) of duplicate wells assayed on at least two separate occasions, and then expressed as ELISA units per mg protein. The results showed that each tablet contained an average of 343 ELISA units per mg of protein. Because we have evidence (unpublished) that antibodies to the O-, H- and colonization factor antigens of ETEC all contribute to protection against infection, we did not assay antibody titers to any of these factors individually.

### Infection and monitoring of volunteers

#### Selection and medical screening

Prospective volunteers were recruited from a population of medical students or subjects with university level education. They were carefully screened to ensure that they were in good physical and mental health. Screening consisted of a medical history, physical examination, urinalysis, complete blood count and a chemistry panel including measurement of aspartate transaminase, alanine aminotransferase, glucose, creatinine, urea and serum electrolytes. Blood was tested for antibodies to HIV and hepatitis C virus and for hepatitis B surface antigen. A stool specimen was examined for ova and parasites and investigated for *E. coli* O78, *Campylobacter, Salmonella* and *Shigella* species as described below.

Inclusion criteria were: healthy adult aged 18–40 years with a normal medical history, physical examination, urine dipstick, blood count and chemistry panel. Exclusion criteria were: (i) a history of immunodeficiency, cardiovascular disease, respiratory disease, endocrine disorder, renal disease, liver disease, gastrointestinal disease, disorder of the reticuloendothelial system, neurological illness, psychiatric disorder or drug or alcohol abuse; (ii) a history of allergy to milk or ciprofloxacin; (iii) a history of ETEC vaccination or an episode of travelers' diarrhea in the past year; (iv) a history of antibiotic therapy during the 7 days before challenge; (v) pregnancy; (vi) positive HIV antibody test; (vii) positive hepatitis B surface antigen or hepatitis C antibody test; (viii) stool microscopy or culture positive for a known enteric pathogen.

#### Study design (Figure 1)

Two separate studies were conducted at the Outpatients Department of the Central Teaching Hospital of the University of Warsaw Medical School. The first study was designed to test the efficacy of BCP tablets given with sodium bicarbonate. The second study was designed to evaluate the dose response and efficacy of BCP tablets taken without sodium bicarbonate. The sample size in each group was based on published studies of challenges of volunteers with the same ETEC strain [9,15,16].

**Figure 1:**
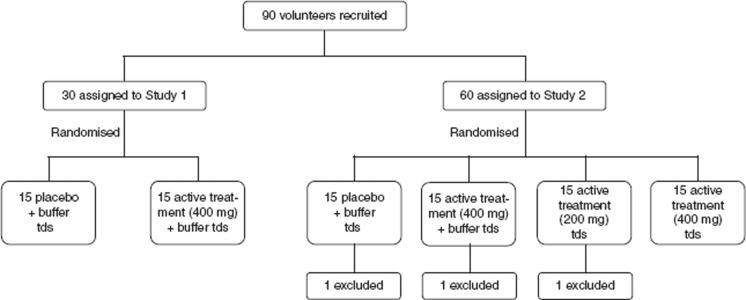
Study design.

Days 1–7 of both studies included the introduction of screened volunteers to the facilities, a complete explanation of the study design and procedures, and the elicitation of informed consent. Volunteers also commenced a diary that noted the date and time of each bowel movement, a score for the type of bowel movement (grade 1 – firm; grade 2 – loose; grade 3 – thick liquid; grade 4 – opaque watery; grade 5 – rice water), and any abdominal cramps or pain. On days 8–14 the challenge experiments were carried out. Volunteers were randomly assigned to placebo or treatment groups in a double-blind manner. The results of both studies were fully analyzed before the treatment group of the participants was known. The clinical protocols for the studies were approved by the Bioethics Committee of the Warsaw Chamber of Physicians (Resolution number 7/00), and the procedures followed were in accordance with the ethical standards of this Committee and with the Helsinki Declaration of 1975, as revised in 1983.

#### Study 1

Thirty volunteers were randomized into two groups of 15 by drawing lots. Investigators and participants remained unaware of the identity of the groups until the after study had been completed and the results had been analyzed. Volunteers in each group were given either two BCP or two placebo tablets three times a day from coded containers together with 100 ml of sodium bicarbonate solution (pH 7.2–7.4) under direct supervision before meals for 7 days. Participants took the seventh dose of tablets before lunch on the third day. Immediately after lunch all participants were challenged with ∼10^9^ colony-forming units of *E. coli* H10407 suspended in 20 ml of sodium bicarbonate. The challenge strain was chosen because it represents a common cause of travelers' diarrhea and has previously been used to infect volunteers [9,15,16]. It was administered at a dose expected to cause diarrhea in ∼90% of susceptible people.

Participants continued to take the tablets that were assigned to them throughout the study. Each participant was seen by clinical staff three times a day, and maintained a diary of their bowel movements and gastrointestinal symptoms. Diarrhea was defined as the passage of two or more unformed (grades 3–5) stools during a 48-h period within 72 h of the challenge. Abnormal bowel movements (grades 3–5) were cultured for *Salmonella, Shigella, Campylobacter* and *E. coli* O78.

Volunteers who developed diarrhea during the trial were given oral glucose and electrolyte replacement and then, 24 h after reporting diarrhea, were treated with 500 mg ciprofloxacin twice daily for 5 days. All other participants were given ciprofloxacin 500 mg twice daily for 5 days beginning 5 days after the challenge to eradicate the challenge strain. All participants were examined by medical personnel once weekly for 3 weeks after the conclusion of the trial when a final bacteriological examination of fecal samples was performed to ensure that the challenge strain had been eradicated.

#### Study 2

Sixty volunteers were assigned at random to one of four groups as for Study 1. The experimental methods were broadly the same as for Study 1, except that the treatment groups were as follows:

Group 1: two tablets of placebo with 100 ml sodium bicarbonate solution, three times daily; Group 2: two tablets of active product with 100 ml sodium bicarbonate solution, three times daily; Group 3: one tablet of active product, three times daily, and Group 4: two tablets of active product, three times daily.

Participants in both studies were permitted to leave the facility during the course of the study, but returned at least three times daily to report any symptoms and receive their medication.

#### Deviations from study protocol

Three participants in Study 2 (one from each of groups 1, 2 and 3) were excluded from the analysis because they were asymptomatic carriers of an O78 *E. coli* strain at the start of study. None of these volunteers developed diarrhea after challenge with *E. coli* H10407.

### Bacteriological analysis

Bacteriological examination of stools before, during and after challenge was performed at the National Institute of Hygiene (SANEPID), Warsaw, to exclude *Salmonella, Shigella* and *Campylobacter* species. All abnormal stools were plated onto Levine's eosin–methylene-blue agar (BD, Franklin Lakes, NJ, USA). Five colonies with a metallic sheen typical of *E. coli* were picked and tested for agglutination with O78 antiserum (Statens Serum Institute, Copenhagen, Denmark). Agglutinable colonies were transferred to Trypticase soy agar slants and transported to Melbourne, where the isolates were retested with O78 antiserum and investigated by PCR for the presence of the genes for the heat-stable and heat-labile enterotoxins of ETEC, as described previously [17].

### Statistical analysis

The results were analyzed for statistical differences between groups by using Fisher's exact test or Student's *t*-test as appropriate. Two-tailed values of *p* < 0.05 were taken to indicate statistical significance. Treatment efficacy was calculated using the formula: 1 – relative risk, expressed as a percentage.

## Results

None of the 90 volunteers who received BCP (*n* = 60) or lactose (*n* = 30) developed any adverse symptoms before ingesting *E. coli* H10407. Ingestion of the ETEC challenge strain by volunteers led to clinically evident infection in both studies, with attack rates of diarrhea in the placebo groups in Studies 1 and 2 of 73% and 86%, respectively (Tables I and II). Tablets containing BCP afforded statistically significant protection (*p* < 0.05) against the development of diarrhea in all treatment groups. Importantly, there was no significant difference in the protection against diarrhea afforded by BCP given with or without sodium bicarbonate buffer to protect the colostrum from stomach acid (Table II). Reports of abdominal pain were significantly less in all three treatment groups who received 400 mg of BCP (Tables I and II). When diarrhea did occur, the number of diarrheal stools in the placebo and treatment groups was similar. Because ciprofloxacin was administered to all participants soon after they developed diarrhea, our ability to compare illness severity in the treatment and placebo groups was limited.

**Table I:** Summary of results of study 1: prophylactic efficacy of hyperimmune bovine colostrum powder (400 mg doses) and placebo against infection with enterotoxigenic *E. coli* strain, H10407

	Treatment group		
		
	Placebo	Colostrum	p*
Number of volunteers	15	15	
Number of volunteers with diarrhea	11 (73%)	1 1(73%)	0.0005
Number of diarrheal stools/volunteer (mean ± SEM)	4.4 ± 0.9	0.4 ± 0.4	0.0004
Mean number of diarrheal stools per volunteer with diarrhea (mean and range)	6 (2–8)	6(6)	NS
Abdominal pain	5 (33%)	0 (0%)	0.04
ETEC H10407 isolated from feces after challenge	15 (100%)	12 (80%)	NS

* Fisher's exact test or Student's *t*-test (two-tailed) as appropriate. NS, not significant.

**Table II:** Summary of results of study 2: prophylactic efficacy of different doses of hyperimmune bovine colostrum powder with or without sodium bicarbonate buffer against infection with enterotoxigenic *E. coli* strain, H10407

	Treatment group			
	
	Group 1: Placebo tid	Group 2: Colostrum 400 mg tid + buffer	Group 3: Colostrum 200 mg tid	Group 4: Colostrum 400 mg tid
Number of volunteers	14	14	14	15
Number of volunteers with diarrhea	12 (86%)	2 (14%), *p* = 0.0004*	5 (36%), *p* = 0.02	3 (20%), *p* = 0.007
Number of diarrheal stools/volunteer (mean ± SEM)	3.9 ± 0.8	0.5 ± 0.3, *p* = 0.0005	1.8 ± 0.8, *p* = 0.07	0.9 ± 0.5, *p* = 0.003
Mean number of diarrheal stools per volunteer with diarrhea (mean and range)	5 (3-10)	3.5 (3-4)	5 (2-7)	4.7 (2-7)
Abdominal pain	5 (36%)	0 (0%), *p* = 0.04	2 (14%), *p* = 0.04	0 (0%), *p* = 0.02
ETEC H10407 isolated from feces after challenge	12 (86%)	14 (100%)	14 (100%)	12 (80%)

* All tests of significance compared the results of active treatment with the placebo group and were calculated by using Fisher's exact test or Student's *t*-test (two-tailed) as appropriate.

BCP had no effect on the viability of the challenge strains, which was recovered from the feces of almost all of the study participants, regardless of the treatment they received (Tables I and II). The strains were eradicated by the treatment with ciprofloxacin, and all volunteers had cleared the organism and were free from symptoms at the end of the treatment-free follow-up period.

## Discussion

This study has demonstrated that the tablets containing BCP with high titers of antibodies against ETEC can provide protection against the development of diarrhea following the ingestion of a homologous ETEC strain. Importantly, the efficacy of the treatment was not significantly reduced by the omission of sodium bicarbonate buffer, which would be expected to protect the BCP preparation from stomach acid. The finding that bicarbonate buffer was not needed for this preparation may be explained by the fact that the proteins in cow's milk can provide sufficient buffering on their own [18]. Further examination of this finding has led to the discovery that non-antibody proteins in colostrum can protect the antibodies themselves and potentially other delicate moieties in acid environments such as the stomach. This finding is further described in Australian Patent 2003212098 and US Patent 2005/0175597 (http://www.freepatentsonline.com/y2005/0175597.html).

The studies reported here emulated the ability of a tablet formulation of BCP to prevent diarrhea in someone eating a contaminated meal. The results showed that when taken before eating or drinking contaminated food or drink, the tablet formulation can protect travelers from symptomatic infection with ETEC of a homologous serotype. The results are consistent with the hypothesis that the tablet formulation allowed delivery of protective antibodies to the small intestine, which is the site of infection with ETEC [3]. As the vaccine used to immunize cattle did not contain ETEC enterotoxins and the BCP preparation did not contain antibodies to these toxins (data not shown), the anti-ETEC activity of the BCP was most likely due to its ability to interfere with the binding of ETEC cells to the intestinal mucosa [3]. This hypothesis is supported by the fact that the BCP preparation did not interfere with the viability of the challenge strain.

As in other studies, which have investigated the use of orally administered hyperimmune bovine colostrum or milk immunoglobulin for a variety of conditions [19–24], no adverse effects were ascribed to the BCP preparation that was used in this study. Published reports of the use of hyperimmune bovine colostrum or milk immunoglobulin to prevent or manage GIT infections caused by rotavirus, *Cryptosporidium, Clostridium difficile, Shigella flexneri, Helicobacter pylori* and ETEC have provided conflicting results [9,10,19–28]. Some studies failed to demonstrate a protective effect of these preparations [10,27,28], whereas others showed that they can provide protection against the development of clinical manifestations of infection or significantly decrease their severity [9,19,20,22,24,25]. The doses of hyperimmune colostrum concentrates in some of these studies were as high as 10 g per day, in contrast to our study which used 0.6–1.2 g of a similar extract per day.

Studies using colostral proteins to reduce gastrointestinal damage after non-steroidal anti-inflammatory drug therapy have pointed toward the presence of intrinsic growth factors in bovine colostrum (such as epidermal growth factor), and broad-spectrum antimicrobial factors (such as lactoferrin) that may have a beneficial effect on the health of the intestinal mucosa [29–31]. Possibly, the success of tablets made from BCP in protecting against challenge with ETEC was partly due to the effect of increased mucosal resistance caused by some of these bioactive factors in addition to the specific anti-ETEC antibodies. It is most unlikely, however, that the action of these other bioactive factors alone can explain our findings, since several studies have demonstrated that while non-immune colostrum or milk extract may assist recovery from gut damage, it has little effect on the prevention of infectious diarrhea [9,20,24].

In conclusion, this study has demonstrated that a novel tablet formulation of hyperimmune BCP containing antibodies against 14 different ETEC strains can significantly reduce the risk of diarrhea following exposure to a homologous ETEC strain. The beneficial effect of BCP was independent of the concurrent use of a sodium bicarbonate buffer to aid passage of the active product through the stomach. These results indicate that practicable formulations of hyperimmune colostrum can reduce the risk of ETEC-induced diarrhea in travelers to high-risk destinations.
